# Photo-reduced Cu/CuO nanoclusters on TiO_2_ nanotube arrays as highly efficient and reusable catalyst

**DOI:** 10.1038/srep39695

**Published:** 2017-01-10

**Authors:** Zhao Jin, Chang Liu, Kun Qi, Xiaoqiang Cui

**Affiliations:** 1 Department of Materials Science, State Key Laboratory of Automotive Simulation and Control, and Key Laboratory of Automobile Materials of MOE, Jilin University, Changchun 130012, People’s Republic of China

## Abstract

Non-noble metal nanoparticles are becoming more and more important in catalysis recently. Cu/CuO nanoclusters on highly ordered TiO_2_ nanotube arrays are successfully developed by a surfactant-free photoreduction method. This non-noble metal Cu/CuO-TiO_2_ catalyst exhibits excellent catalytic activity and stability for the reduction of 4-nitrophenol (4-NP) to 4-aminophenol (4-AP) with the presence of sodium borohydride (NaBH_4_). The rate constant of this low-cost Cu/CuO based catalyst is even higher than that of the noble metal nanoparticles decorated on the same TiO_2_ substrate. The conversion efficiency remains almost unchanged after 7 cycles of recycling. The recycle process of this Cu/CuO-TiO_2_ catalyst supported by Ti foil is very simple and convenient compared with that of the common powder catalysts. This catalyst also exhibited great catalytic activity to other organic dyes, such as methylene blue (MB), rhodamine B (RhB) and methyl orange (MO). This highly efficient, low-cost and easily reusable Cu/CuO-TiO_2_ catalyst is expected to be of great potential in catalysis in the future.

Nitroaromatic compounds are very important industrial chemicals nowadays and are widely used in the manufacture of dyes, plastics, pesticides, and explosives^1^. The release of these compounds in natural water will cause serious environmental pollution since that most of them are considered as potential toxicity to organisms^2^. 4-nirtophenol (4-NP) is one of the most common nitroaromatic compounds in industrial effluents and has been classified as priority pollutant by the US Environmental Protection Agency^3^,^4^. However, 4-NP is very stable in the environment and hardly to be biodegraded. An efficient and environment friendly method to remove them from waste water is the direct reduction of 4-NP in the presence of NaBH_4_ and catalyst to 4-aminophenol (4-AP), which is a very important intermediate for the manufacture of analgesic and antipyretic drugs^5^. It is highly desirable to find an efficient and eco-friendly catalyst for this reduction.

Noble metal nanoparticles such as Au, Ag, Pt, Pd and their alloys are commonly used as catalysts for the reduction of 4-NP in industry because of the high catalytic activity^6^,^7^,^8^,^9^,^10^,^11^,^12^,^13^,^14^, but the expensiveness and rareness of noble metals limit their extensive applications in catalysis. Design and fabrication of non-noble metal catalyst with high activity is becoming increasingly important. Copper (Cu) based composites are receiving more and more attention due to their relatively low cost, large abundance and great catalytic activity^15^,^16^,^17^,^18^. Recent reports have shown that Cu^15^,^19^,^20^, Cu_2_O^16^,^21^,^22^ and CuO^17^,^23^,^24^ nanostructures exhibited excellent catalytic activities comparable to or even higher than that of noble metals. But the aggregation of metal nanoparticles during reaction usually leads to the rapid decrease of catalytic activity^25^,^26^ and the recycle process of these small catalysts by repeating centrifuging and washing is time-consuming and inefficient^15^. These Cu-based catalysts are still suffering from the stability and reusability problems. It is highly demanded to design a nano-catalyst with high activity that can prevent the aggregation and also be easily recycled.

Herein, we fabricate Cu/CuO-TiO_2_ catalyst by decorating non-noble Cu/CuO nanoclusters on highly ordered TiO_2_ nanotube (NT) arrays through a surfactant-free photoreduction method^27^,^28^. This novel catalyst exhibits several advantages in catalytic application: (1) cost-efficient. These Cu/CuO nanoclusters based catalyst are much cheaper than noble metals; (2) high activity. This catalyst shows excellent catalytic activity towards 4-NP attributed to the sufficient “clean” surfaces of Cu/CuO nanoclusters provided by the surfactant-free photoreduction method; (3) great stability. This Cu/CuO-TiO_2_ catalyst exhibits excellent stability because the *in-situ* formed nanoclusters are firmly combined with the TiO_2_ NT substrate, which prevents the aggregation and loss of the nanoclusters effectively; (4) easy to recycle. The recycle process of Cu/CuO-TiO_2_ is simple by only removing the TiO_2_/Ti foil supported catalysts out of the reaction media with tweezers and rinsed with DI water; (5) universal. The Cu/CuO-TiO_2_ catalyst also exhibits great catalytic activity to other dyes including methylene blue (MB), rhodamine B (RhB) and methyl orange (MO).

## Materials and Methods

### Materials

The Ti foil was purchased from Sigma-Aldrich (99.7%, 0.127 mm). Ethylene glycol (EG), sodium borohydride (NaBH_4_), copper chloride (CuCl_2_), chloroauric acid (HAuCl_4_), and palladium chloride (PdCl_2_) were from Sinopharm Chemical Reagent Co., Ltd, China. Ammonia fluoride (NH_4_F), 4-NP, MB, RhB, MO, silver nitrate (AgNO_3_) and ethanol were obtained from Beijing Regent Co. China. Deionized (DI) water was used throughout the experiments with a resistivity of 18.2 MΩ cm.

### Instrument

Constant voltage for anodization of Ti was conducted on a SAKO DC power supply. The photoreduction was performed on a 300 W Xe lamp illumination. X-ray diffraction (XRD) data were collected by a D8 advanced Bragg-Brentano diffractometer (Bruker AXS, Germany). Morphologies were characterized by a JEM-6700F (JEOL, Japan) scanning electron microscope (SEM). Transmission electron microscopy (TEM) images were acquired by a JEM-2100F transmission electron microscope (JEOL, Japan). X-ray photoelectron spectroscopy (XPS) data were acquired with an ESCALAB-250 instrument (Thermo Fisher Scientific, USA). The UV-Vis adsorption spectra were recorded on a USB4000 UV–Vis spectrophotometer (Ocean Optics Inc., US).

### Preparation of TiO_2_ Nanotube

Two-step anodization of Ti foil was carried out to fabricate the highly ordered TiO_2_ nanotube (NT) arrays^29^,^30^. Firstly, the Ti foil was degreased by sonication in acetone and ethanol, followed by rinsing with water and drying with nitrogen. Then, anodization was carried out using a conventional two-electrode system. Ti foil was working as anode and Pt gauze as cathode. The electrolyte was ethylene glycol including 0.3 wt% NH_4_F and 2 vol% DI water. The temperature of the reaction was kept at 25 °C by a circulating water bath. The Ti foil was first anodized under a constant voltage of 60 V for 1 h, leading to the formation of irregular TiO_2_ NT arrays. These irregular arrays were ultrasonically removed in DI water and the same Ti foil was anodized again under 40 V for 1 h. After this second anodization, highly ordered and regular TiO_2_ NT arrays were formed on top of the Ti foil. Compared to the irregular NT arrays, these two-step-fabricated highly ordered TiO_2_ NT arrays can provide greater absorption of incident light^31^ and offer more suitable nucleation sites for metal nanoparticles during the photoreduction as discussed in our previous work^30^. The anodized TiO_2_ substrates were rinsed with ethanol, dried with pure nitrogen and finally annealed in air at 480 °C for 3 h with a heating rate of 5 °C/min^30^,^32^.

### Cu/CuO Nanoclusters Decoration

*In-situ* surfactant-free photoreduction method was carried out for the decoration of “clean” Cu/CuO nanoclusters on top of the ordered TiO_2_ NT arrays^27^,^28^. First, different concentration of CuCl_2_ aqueous (0.02 mM, 0.1 mM and 0.5 mM) contained 5 vol% ethanol were prepared and saturated with N_2_. Then, TiO_2_ NT substrates were soaked in these CuCl_2_ aqueous for 30 min for the absorption of Cu^2+^ onto the surface. Finally, it was irradiated *in-situ* with a 300 mW/cm^2^ white light for 90 min to reduce the absorbed Cu^2+^ into Cu by the photocatalysis of TiO_2_. The newly formed Cu nanoclusters are very easy to be oxidized exposed in air and thin CuO passivation layers are evenly formed on the surfaces. The as-prepared Cu/CuO-TiO_2_ samples were rinsed with DI water, dried with nitrogen flow. The sample prepared in 0.02 mM, 0.1 mM and 0.5 mM CuCl_2_ solution is denoted as C-0.02, C-0.1 and C-0.5, respectively.

Other noble metal nanoparticles, such as Au, Ag and Pd were also prepared onto the TiO_2_ NT arrays for comparison using the same photoreduction method in 0.1 mM HAuCl_4_, AgNO_3_ and PdCl_2_, respectively.

### Catalysis Procedures

**Figure 1:**
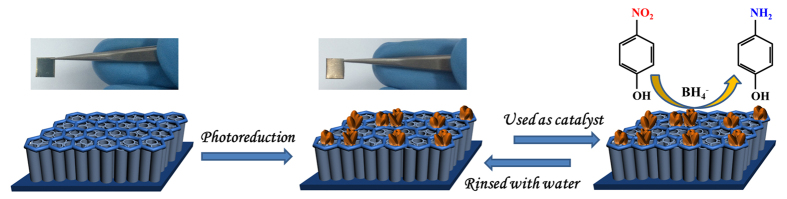
Fabrication and reusability schematic of Cu/CuO-TiO_2_ catalyst for the 4-NP reduction with NaBH_4_.

The reduction of 4-NP to 4-aminophenol (AP) in the presence of NaBH_4_ was performed to test the catalytic activity of Cu/CuO-TiO_2_ catalyst. Typically, the reaction was carried out in a quartz cuvette at room temperature under stirring and monitored by a UV-vis spectrophotometer. 0.25 ml 4-NP aqueous solution (1 mM) and 0.25 ml freshly prepared NaBH_4_ (100 mM) were mixed with 2 ml DI water. Subsequently, a 0.6 × 0.6 cm^2^ Cu/CuO-TiO_2_ catalyst was soaked in the solution and the absorption spectrum was recorded every 0.5 minute. Excess of NaBH_4_ was used to eliminate the influence of BH_4_^−^ on the reaction. In the recycle test, the catalyst was easily taken out of the solution by tweezers, rinsed with DI water, dried with pure N_2_ and reused for the second cycle directly. Figure 1 shows the fabrication and reusability schematic of the catalyst for the 4-NP reduction with NaBH_4_. Reduction of other organic dyes, such as MB, RhB and MO were also performed using Cu/CuO-TiO_2_ as catalyst in the same condition excepted for the concentration, which were adjusted to 0.1 mM to avoid the over-ranging during UV-vis spectrophotometer monitored.

## Results and Discussion

### Characterization

**Figure 2:**
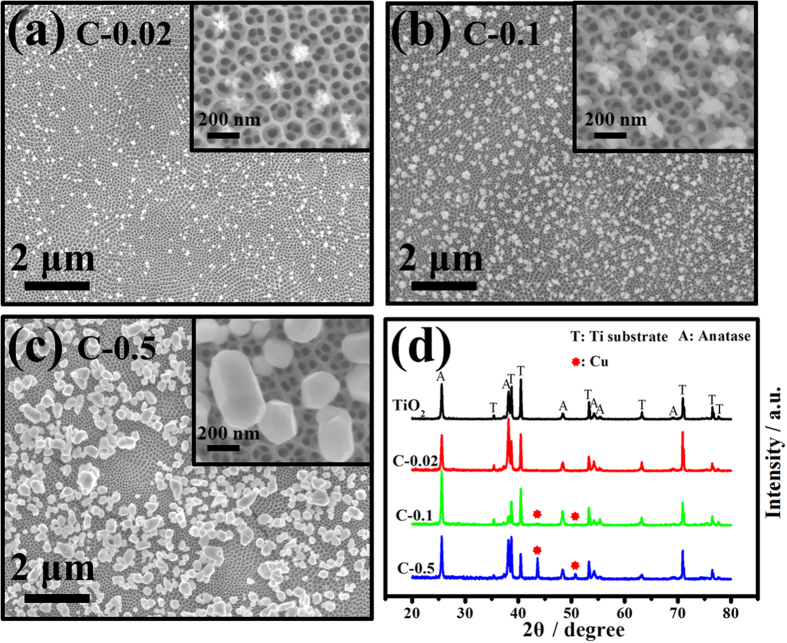
SEM images of the fabricated catalysts. (**a**) C-0.02; (**b**) C-0.1; (**c**) C-0.5 and (**d**) their corresponding XRD patterns compared with TiO_2_ nanotube arrays. Insets show the high magnification images of the catalysts.

SEM and XRD characterizations were firstly carried out to investigate the morphologies and compositions of Cu/CuO-TiO_2_ catalysts fabricated in different concentration of CuCl_2_. SEM images of TiO_2_ nanotube arrays without Cu/CuO decoration are shown in Supplementary Fig. S1. Figure 2a,b and c are the top view SEM images of C-0.02, C-0.1 and C-0.5, respectively. The concentration of CuCl_2_ shows significant influence on the morphology of the catalyst. The size distributions of Cu/CuO on these catalysts are shown in Supplementary Fig. S2. For catalyst C-0.02, Cu/CuO nanoclusters with average size of approximate 81 nm are formed and dispersed on top of TiO_2_ substrate after photoreduction in 0.02 mM CuCl_2_ as shown in Fig. 2(a). Increasing the concentration of CuCl_2_ to 0.1 mM led to the formation of larger Cu/CuO nanoclusters with average size of 158 nm consisted of small nanoparticles with size of 50~80 nm. The amount of nanoclusters distributed on TiO_2_ is also increased apparently as in Fig. 2(b). Further increase the concentration of CuCl_2_ to 0.5 mM, Cu/CuO nanocrystal instead of nanoclusters with average size of more than 220 nm are formed in large amount and covered the surface of TiO_2_ NT arrays mostly as observed in Fig. 2(c). The XRD patterns of TiO_2_, C-0.02, C-0.1 and C-0.5 are shown in Fig. 2(d). The diffraction peaks of Ti and anatase^30^,^33^,^34^ are clearly observed on all the samples. No obvious peaks assigned to Cu are observed on TiO_2_ substrate or catalyst C-0.02 with very small size and amount of Cu nanoclusters. Weak diffraction peaks at 43.6° and 50.7° assigned to Cu are observed for catalyst C-0.1. Catalyst C-0.5 shows much clearly diffraction peaks of Cu because of the large size and crystallinity of the Cu^15^. There are no diffraction peaks of copper oxide shown in Fig. 2(d) because of the tiny amount of CuO formed on the surfaces of Cu nanoclusters.

**Figure 3:**
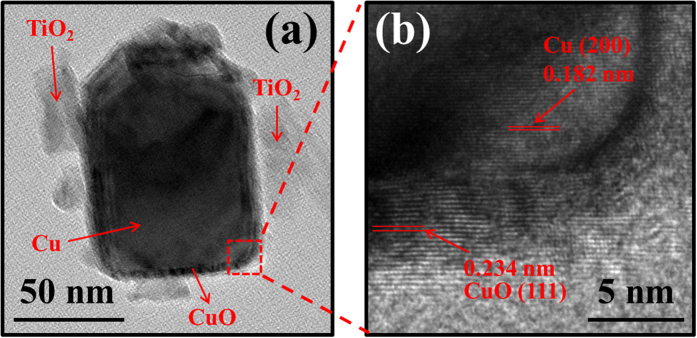
(**a**) TEM image of Cu/CuO nanoparticle obtained from C-0.1; (**b**) High resolution TEM image of the marked region.

Figure 3(a) shows the TEM image of a Cu/CuO nanoparticle scratched off the TiO_2_ nanotube. Slight amount of the residual TiO_2_ are observed around the particle as marked. The size of the nanoparticle is about 70 nm, which is in agreement with the SEM images in Fig. 2(b). A thin layer of CuO on the surface of the nanoparticle is also observed as expected. High resolution TEM image of the marked region is shown in Fig. 3(b) and the crystal lattice fringes of Cu and CuO are clearly observed. The measured lattice spacing inside the nanoparticle is 0.182 nm corresponding to the (200) plane of Cu^19^ and the lattice spacing on the edge of the nanoparticle is 0.234 nm corresponding to the (111) plane of CuO^35^. The TEM characterization certainly proved that the surfaces of Cu were oxidized in air and formed a CuO protective layer with thickness of about 5 nm.

**Figure 4:**
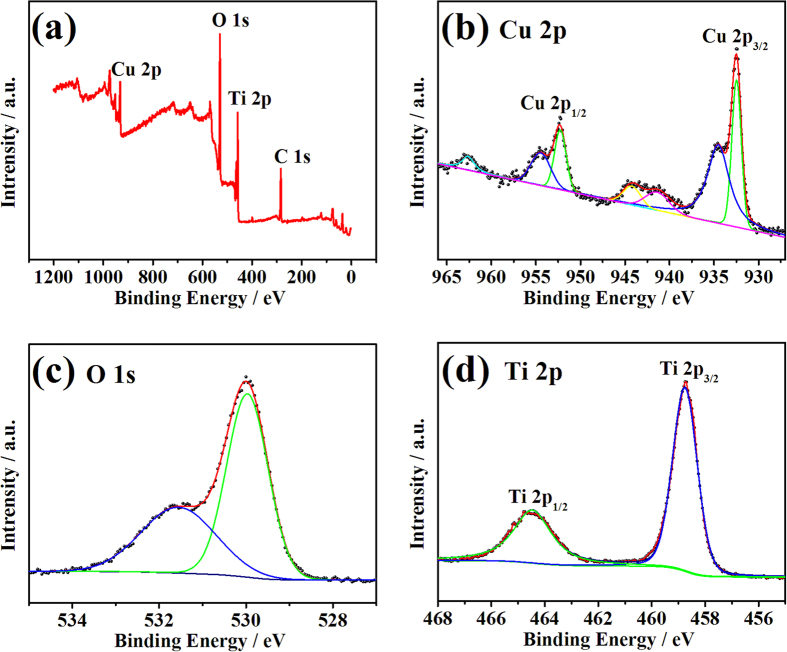
XPS spectra of catalyst C-0.1. (**a**) XPS survey spectrum; (**b**) Cu 2p spectrum; (**c**) O 1 s spectrum and (**d**) Ti 2p spectrum.

XPS measurement was further performed to confirm the surface composition and the elemental chemical states of the catalyst. Figure 4 shows the XPS spectra of C-0.1. The full XPS survey spectrum shown in Fig. 4(a) proves the presence of Cu, O and Ti in the catalyst, which is also confirmed by the electron dispersed spectroscopy (EDS) characterization shown in Supplementary Fig. S3. Cu 2p XPS spectrum is depicted in Fig. 4(b). Major peaks of Cu 2p_3/2_ at 932.5 eV and Cu 2p_1/2_ at 952.3 eV confirmed the existed of metallic copper^20^,^21^,^36^, demonstrated the successful photoreduction of CuCl_2_. Meanwhile, the peaks of Cu 2p_3/2_ at 934.6 eV and Cu 2p_1/2_ at 954.6 eV in combination with the satellite peaks at 941.5 eV and 944.3 eV are typical characteristics of CuO^20^,^36^,^37^, implying the uniformly surface oxidation of Cu nanoclusters exposed in air under ambient conditions. These results are consistent with the TEM characterization. O 1 s spectrum shown in Fig. 4(c) consists of two peaks. The major peak at 530.0 eV corresponds to O^2−^ in CuO and TiO_2_. The secondary peak at 531.6 eV is attributed to the oxygen species adsorbed on the surface^16^,^38^. Ti 2p spectrum is shown in Fig. 4(d). The peaks of Ti 2p_3/2_ at 458.8 eV and Ti 2p_1/2_ at 464.5 eV are consistent with the typical TiO_2_^38^. The result of XPS measurement further proved the existence of Cu/CuO composite on TiO_2_ substrate.

### Catalytic Activity

Reduction of 4-NP to 4-aminophenol (AP) with NaBH_4_ was first carried out to evaluate the catalytic activity of Cu/CuO-TiO_2_. As shown in Supplementary Fig. S4, 4-NP aqueous solution exhibits a strong absorption peak at 316 nm, which remarkably shifted to 400 nm after the addition of NaBH_4_ due to the formation of 4-nitrophenolate ions under alkaline conditions in Fig. S4(a)^26^,^39^. This absorption peak remains unchanged in more than 30 minutes in Fig. S4(b) indicated that the reduction reaction did not proceed without catalyst. Figure S4(c) demonstrated that the reduction is still unable to proceed with only TiO_2_ NT substrate dipped into the solution. After Cu/CuO nanoclusters decoration, the Cu/CuO-TiO_2_ catalysts exhibit great catalytic activity towards 4-NP.

**Figure 5:**
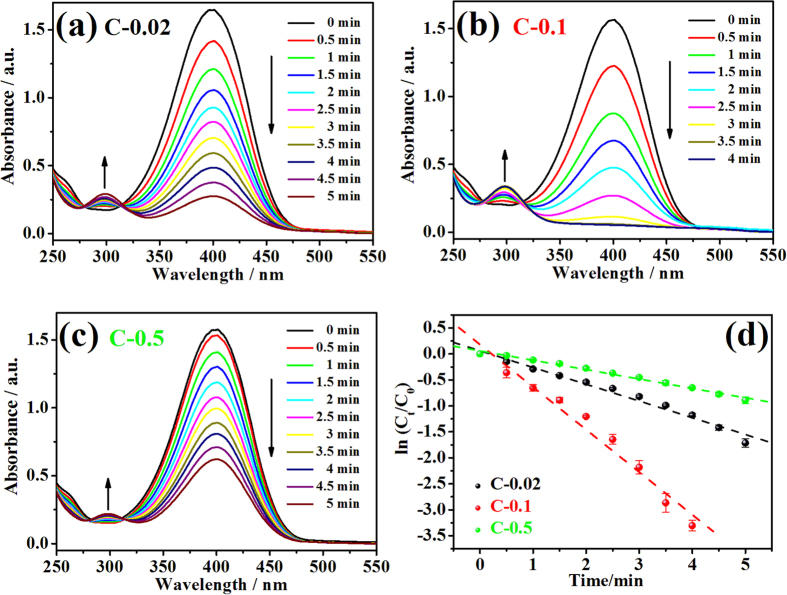
Time-dependent UV-vis absorption spectra for the reduction of 4-NP with (**a**) C-0.02; (**b**) C-0.1; (**c**) C-0.5; and (**d**) the corresponding plots of ln (C_t_/C_0_) versus reaction time.

The time-dependent UV-vis absorption spectra for the reduction of 4-NP with C-0.02, C-0.1 and C-0.5 were compared in Fig. 5(a~c). The reduction started immediately after the immersion of the catalysts with no need of induction time and the absorption peak of 4-NP at 400 nm gradually decreased as the reaction proceeded. Meanwhile, new absorption peak of 4-AP at 300 nm appears and gradually increases. Isosbestic point between these two absorption peaks is also shown in the absorption spectra, indicating the clean conversion from 4-NP to 4-AP without any byproducts^19^. Catalyst C-0.1 exhibits the best catalytic activity for 4-NP, of which the reduction reaction is completed within 3.5 minutes as shown in Fig. 5(b). For catalyst C-0.02 and C-0.5, it takes more than 5 minutes to complete the reaction as shown in Fig. 5(a) and (c). The amount of NaBH_4_ in this system is excessive to ensure the reaction followed pseudo-first-order kinetics with respect to 4-NP only^19^. Therefore, the kinetics can be described as −kt = ln (C_t_/C_0_), where k is the first-order rate constant, t is the reaction time, C_t_ and C_0_ stands for the 4-NP concentrations at time t and 0^15^. The corresponding linear relationships of ln (C_t_/C_0_) versus reaction time are shown in Fig. 5(d) and the rate constant, k, is calculated from the slopes of the fitted straight lines. The highest rate constant obtained from C-0.1 is 13.6 × 10^−3^ s^−1^. This excellent catalytic activity is attributed to the great amount of Cu/CuO nanoclusters evenly dispersed on TiO_2_ NT arrays, providing sufficient “clean” surfaces as active sites obtained from the green and surfactant-free photoreduction method. For catalyst C-0.02 and C-0.5, the k values are 5.4 × 10^−3^ s^−1^ and 3.0 × 10^−3^ s^−1^, respectively. The decrease of rate constant for C-0.02 is because the total amount of Cu/CuO nanoclusters decorated on TiO_2_ NT arrays is small, which provides limited active sites for 4-NP reduction compared with C-0.1. For C-0.5, although the amount of Cu/CuO is large enough to cover most of the TiO_2_ surface, the formation of Cu/CuO nanocrystal with large size reduces the surface area exposed to 4-NP and decrease the catalytic activity dramatically. Meanwhile, the turnover frequency (TOF) is another important parameter for catalysis and the TOF of C-0.1 is 115 h^−1^. The details of TOF calculation is provided in the end of Supplementary.

**Figure 6:**
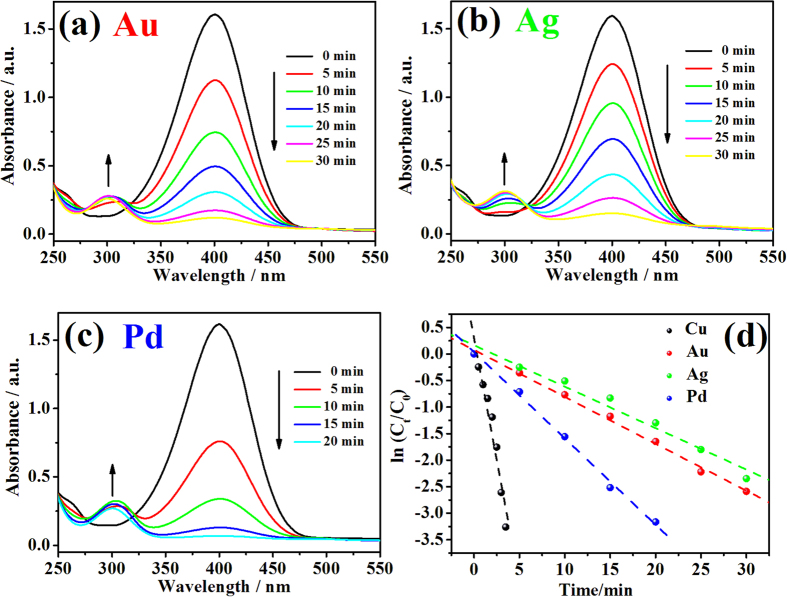
Time-dependent UV-vis absorption spectra for the reduction of 4-NP with different catalysts. (**a**) Au-TiO_2_; (**b**) Ag-TiO_2_; (**c**) Pd-TiO_2_. (**d**) The corresponding plots of ln (C_t_/C_0_) of different catalysts versus reaction time.

Different noble metal nanoparticles were decorated onto the TiO_2_ NT arrays using the same photoreduction method and their catalytic activities were compared with catalyst C-0.1. SEM characterizations shown in Supplementary Fig. S5(a–c) confirm the successful decoration of Au, Ag and Pd nanoparticles on TiO_2_, revealing that this surfactant-free method is suitable for various metals^28^. Figure 6(a–c) show the time-dependent UV-vis absorption spectra of 4-NP reduction with Au-TiO_2_, Ag-TiO_2_ and Pd-TiO_2_ as catalyst, respectively. The corresponding plots of ln (C_t_/C_0_) versus reaction time shown in Fig. 6(d) indicates that the reduction of 4-NP by these noble metal catalysts also followed pseudo first-order kinetics. The calculated rate constants are 1.5 × 10^−3^ s^−1^ (Au-TiO_2_), 1.3 × 10^−3^ s^−1^ (Ag-TiO_2_) and 2.7 × 10^−3^ s^−1^ (Pd-TiO_2_) as listed in Supplementary Table S1. The rate constant of C-0.1 is higher than that of these noble metal nanoparticles fabricated in the same photoreduction method. Other metal nanostructures decorated bulk substrates as reusable catalysts for 4-NP reduction mentioned in references are also listed in Supplementary Table S1^8^,^10^,^26^,^36^. As observed, the rate constant of C-0.1 in this article is also comparable to or higher than that of these catalysts, especially the noble metal catalysts. Considering the low-cost of Cu, this Cu/CuO-TiO_2_ catalyst shows certain superiority in practical applications.

### Reusability and Universality

**Figure 7:**
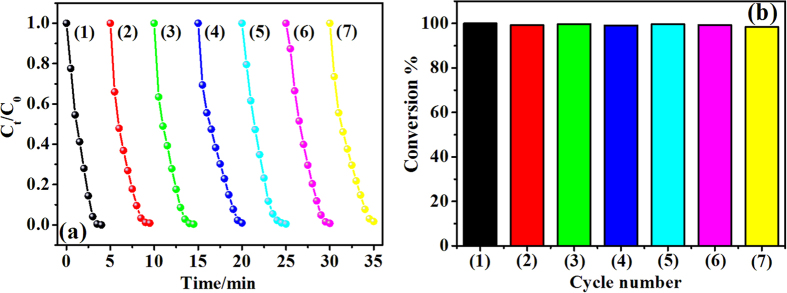
Reusability of the C-0.1 catalyst for the reduction of 4-NP. (**a**) Plots of C_t_/C_0_ versus reaction time for 7 reaction cycles and (**b**) the corresponding conversions of 4-NP.

Reusability and stability are very important aspect of catalysts for their practical applications. To investigate the reusability of our catalyst, same C-0.1 catalyst was used repeatedly up to 7 times for the reduction of 4-NP. The plots of C_t_/C_0_ versus reaction time are shown in Fig. 7(a). The catalytic activity of C-0.1 remains almost unchanged during the reusability test and the reduction reaction can still be completed within 5 minutes even for the seventh cycle. The corresponding conversion for each cycle after 5 minutes remains above 99% as shown in Fig. 7(b), which maintained very well compares with those in refs 15, 19, 25 and 26. This great stability of the catalytic in recycling is attributed to the firmly contacted between Cu/CuO nanoclusters and TiO_2_ NT arrays because the nanoclusters are *in-situ* photo-reduced and growth on TiO_2_ substrate instead of loading after formation, which prevent the aggregation or loss of the nanoclusters. It is worth noting that the recycle process of common powder catalysts by repeating centrifuging, washing and long-time drying in oven is very inefficient and costly for practical applications. However, the Cu/CuO-TiO_2_ catalyst we introduce in this work grows directly on a Ti foil, which makes the recycle of our catalyst very simple and convenient. All you need to do is tweezing the Ti foil out of the solution, rinsing with DI water and drying with N_2_. This whole recycling process only cost few minutes in total. All these properties indicate that this Cu/CuO-TiO_2_ catalyst is very stable and reusable in applications.

**Figure 8:**
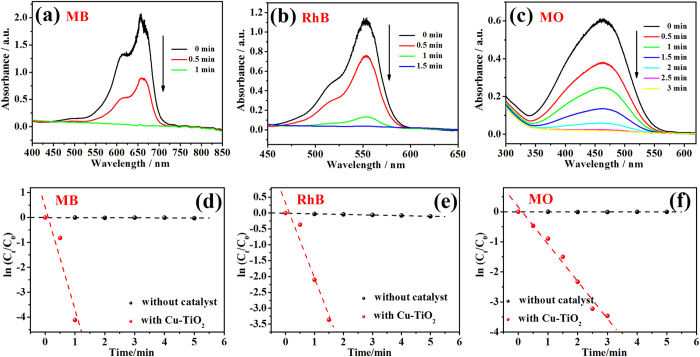
Time-dependent UV-vis absorption spectra for the degradation of (**a**) MB; (**b**) RhB and (**c**) MO with catalyst C-0.1. The corresponding plots of ln (C_t_/C_0_) versus reaction time for (**d**) MB; (**e**) RhB and (**f**) MO.

To examine the universality of Cu/CuO-TiO_2_ as catalyst for other dyes, MB, RhB and MO were also chosen as test targets to investigate the catalytic activity of C-0.1^15^,^40^. Before the addition of catalyst C-0.1, the absorption peak of MB (665 nm), RhB (553 nm) and MO (464 nm) maintains unchanged or very slight decrease in the presence of NaBH_4_ and the corresponding time-dependent UV-vis absorption spectra are given in Supplementary Fig. S6(a–c). After the immersion of the catalyst C-0.1, the absorption intensity decreased very fast and all the reactions are completed in few minutes as shown in Fig. 8(a–c), which indicates that catalyst C-0.1 also exhibits great catalytic activity towards these organic dyes. The corresponding plots of ln (C_t_/C_0_) versus reaction time of these dyes are shown in Fig. 8(d–f). The good linear correlation confirmed that these reactions still followed pseudo-first-order kinetics. The rate constant calculated from the slopes are 68.5 × 10^−3^ s^−1^ (MB), 31.5 × 10^−3^ s^−1^ (RhB) and 20.7 × 10^−3^ s^−1^ (MO), respectively.

## Conclusion

In summary, a highly efficient and reusable Cu/CuO-TiO_2_ catalyst is fabricated using a green method of photoreduction. This non-noble metal catalyst exhibits excellent catalytic activity towards the reduction of 4-NP and other different organic dyes, which is attributed to the large amounts of exposed clean surface provided by the evenly dispersed Cu/CuO nanoclusters. The catalytic activity of this Cu/CuO-TiO_2_ catalyst is even higher than that of the noble metal-TiO_2_ catalyst prepared in the same method. The activity towards 4-NP remains almost unchanged for 7 cycles of the reduction because of the firm connection between the *in-situ* formed nanoclusters and TiO_2_ NT arrays. This low-cost Cu/CuO based catalyst is also very convenient for recycling compared with the complicated and costly recycle process for common powder catalysts. This novel Cu/CuO-TiO_2_ catalyst is expected to replace noble metals as a low-cost, highly efficient and easily reusable catalyst in catalytic applications.

## Additional Information

**How to cite this article**: Jin, Z. *et al*. Photo-reduced Cu/CuO nanoclusters on TiO_2_ nanotube arrays as highly efficient and reusable catalyst. *Sci. Rep.* **7**, 39695; doi: 10.1038/srep39695 (2017).

**Publisher's note:** Springer Nature remains neutral with regard to jurisdictional claims in published maps and institutional affiliations.

## Supplementary Material

Supplementary Information
