# Supplementary material for: Photo-reduced Cu/CuO nanoclusters on TiO2 nanotube arrays as highly efficient and reusable catalyst
Source: Sci Rep. 2017 Jan 10;7:39695. doi: 10.1038/srep39695 (PMC5223138; doi:10.1038/srep39695)
Supplement: Supplementary Information [file srep39695-s1.pdf]

Supporting information

Photo-reduced Cu/CuO nanoclusters on TiO_2 nanotube arrays as highly efficient and reusable catalyst

Zhao Jin, Chang Liu, Kun Qi, Xiaoqiang Cui*

Department of Materials Science, State Key Laboratory of Automotive Simulation and Control, and Key Laboratory of Automobile Materials of MOE, Jilin University, Changchun 130012, People's Republic of China.

*Corresponding Author：Xiaoqiang Cui (e-mail address: xqcui@jlu.edu.cn)

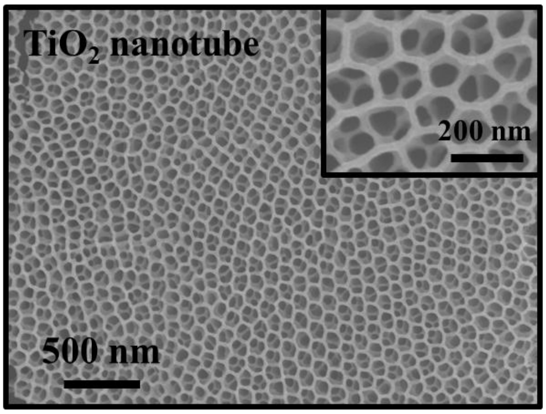

Figure S1. SEM characterization of TiO_2 nanotube arrays without Cu/CuO decoration. Inset shows the high magnification image.

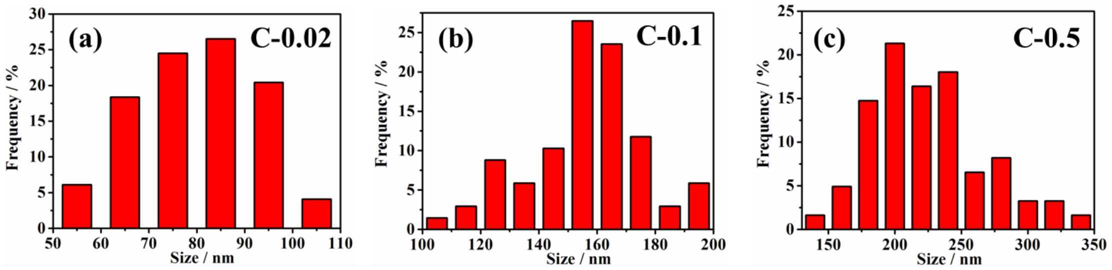

Figure S2. Cu/CuO nanoclusters size distributions of different catalysts: (a) C-0.02, (b) C-0.1 and (c) C-0.5 with average size of 81 nm, 158 nm and 223 nm, respectively.

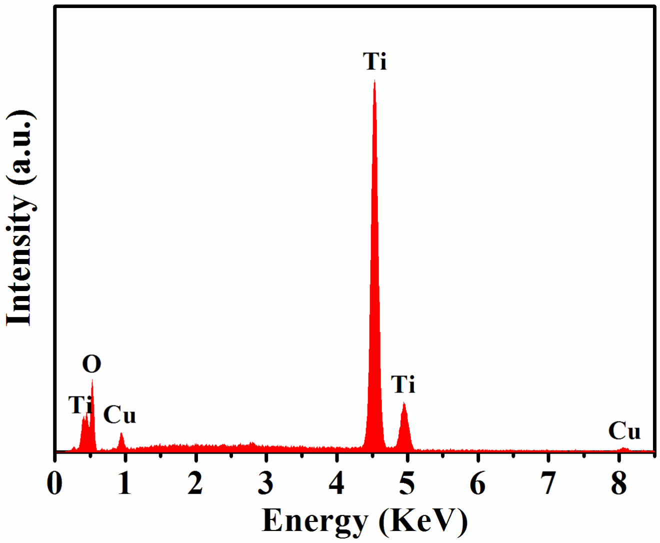

Figure S3. EDS spectrum of catalyst C-0.1.

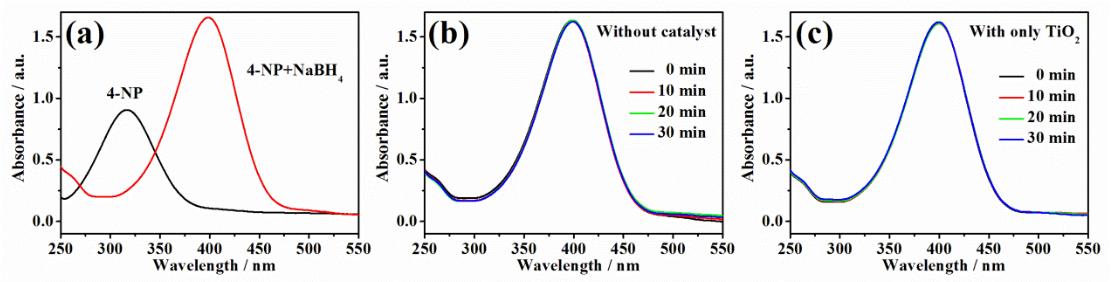

Figure S4. (a) UV-vis absorption spectra of 4-NP with and without $NaBH_4$; Time-dependent UV-vis absorption spectra of 4-NP with $NaBH_4$ but without catalyst (b) and with TiO_2 substrate (c).

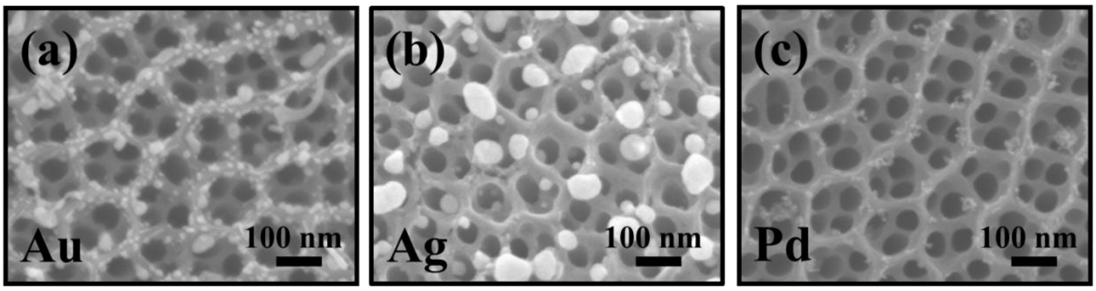

Figure S5. SEM images of TiO_2 substrate with different metal nanoparticles: (a) Au-TiO_2; (b) Ag-TiO_2 and (c) Pd-TiO_2;

Table S1. Comparison of different catalysts for 4-NP reduction.

Catalyst	4-NP(M)	$NaBH_4$(M)	K(s^{-1})	Ref.
C-0.1	1.0×10^{-4}	1.0×10^{-2}	13.6×10^{-3}	This work
Au-TiO_2	1.0×10^{-4}	1.0×10^{-2}	1.5×10^{-3}	This work
Ag-TiO_2	1.0×10^{-4}	1.0×10^{-2}	1.3×10^{-3}	This work
Pd-TiO_2	1.0×10^{-4}	1.0×10^{-2}	2.7×10^{-3}	This work
SiNWAs-Cu	1.0×10^{-4}	1.1×10^{-2}	10.5×10^{-3}	26
Au-PMMA	-	-	1.6×10^{-3}	8
Ag-PMMA	-	-	0.8×10^{-3}	8
Porous Cu	1.0×10^{-3}	1.0×10^{-1}	8.9×10^{-3}	36
Porous Cu/Ag	1.0×10^{-3}	1.0×10^{-1}	19.0×10^{-3}	36
PAu	2.0×10^{-3}	2.0×10^{-1}	0.5×10^{-3}	10
Pd-dendrite/PAu	2.0×10^{-3}	2.0×10^{-1}	1.0×10^{-3}	10

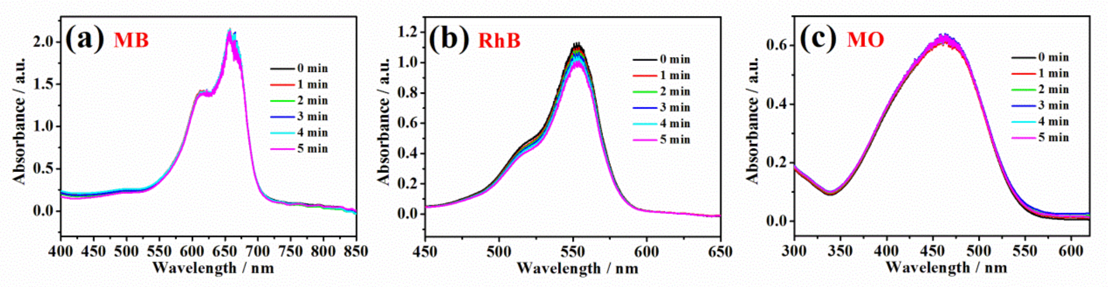

Figure S6. Time-dependent UV-vis absorption spectra for (a) MB; (b) RhB and (c) MO without catalysts.

Calculation of turnover frequency (TOF):

TOF is defined as the moles of reduced 4-NP molecules per mole of catalyst atom per hour. The total mass of Cu on C-0.1 is calculated as 0.0024 mg according to the inductively coupled plasma-optical emission spectrophotometry (ICP-OES). Hence, the molar ratio of 4-NP/Cu is about 6.7. The reaction time for C-0.1 is nearly 3.5 min. As a result, the final TOF is calculated as 115 h^{-1}.
